# Arterial and Venous Pressure Dynamics in Blood Flow Restriction Versus Traditional Strength Training

**DOI:** 10.1111/sms.70029

**Published:** 2025-02-17

**Authors:** Sanghyeon Ji, Alexander Franz, Michaela Vicas, Tobias Boemer, Stefan Luckmann, Michael Behringer, Patrick Wahl

**Affiliations:** ^1^ Section Exercise Physiology German Sport University Cologne Cologne Germany; ^2^ The German Research Center for Elite Sport Cologne Germany; ^3^ Department of Orthopedics and Trauma Surgery University Hospital Bonn Bonn Germany; ^4^ Department of Trauma and Orthopedic Surgery BG Klinikum Ludwigshafen Ludwigshafen Germany; ^5^ Department of Adult Reconstruction ATOS Orthoparc Clinic Cologne Cologne Germany; ^6^ Department of Sports Sciences Goethe University Frankfurt Frankfurt Germany

**Keywords:** arterial hypertension, Kaatsu training, vascular adaptation, venous hypertension

## Abstract

Strength training responses are influenced by sets, repetitions, and mechanical load, whereas Blood Flow Restriction (BFR) training adds the variable of temporarily restricting blood flow via a tourniquet. This has intensified scientific discussions regarding the vascular responses and thereby safety of the BFR method. To address these concerns, we investigated intravascular pressure changes during low‐load (LL‐RT), low‐load with BFR (LL‐BFR‐RT), and high‐load (HL‐RT) exercise. Ten healthy men (26.8 ± 4.59 years) performed unilateral biceps curls to failure in a randomized cross‐over design: (1) LL‐RT (30% 1RM), (2) LL‐BFR‐RT (30% 1RM, 50% LOP), and (3) HL‐RT (75% 1RM). Total workload was significantly higher in LL‐RT (692 ± 251 kg) compared to LL‐BFR‐RT (378 ± 58.7 kg) and HL‐RT (327 ± 65.1 kg, *p* < 0.001). In terms of mean values, LL‐BFR‐RT resulted in higher diastolic and mean arterial pressures during rest periods between sets compared to other conditions (*p* ≤ 0.02). Both LL‐RT and LL‐BFR‐RT led to longer durations spent at increased diastolic (above 90 mmHg, LL‐RT: ~419 s vs. LL‐BFR‐RT: ~356 s vs. Hl‐RT: ~122 s), systolic (above 140 mmHg, LL‐RT: ~437 s vs. LL‐BFR‐RT: ~336 s vs. HL‐RT: ~199 s), and mean arterial pressures (above 107 mmHg, LL‐RT: ~451 s vs. LL‐BFR‐RT: ~384 s vs. HL‐RT: ~168 s) compared to HL‐RT (*p* ≤ 0.028). Relative to total exercise time, LL‐BFR‐RT resulted in higher proportion of time spent at elevated diastolic (above 90 mmHg, LL‐RT: ~56.5% vs. LL‐BFR‐RT: ~68.7% vs. Hl‐RT: ~33.5%) and mean arterial pressures (above 107 mmHg, LL‐RT: ~60.8% vs. LL‐BFR‐RT: ~74.0% vs. HL‐RT: ~45.7%) compared to HL‐RT (*p* ≤ 0.034). Peripheral venous pressure was significantly higher in LL‐BFR‐RT compared to other conditions (*p* < 0.001), with both absolute and relative time spent at higher pressures (above 75 mmHg, LL‐RT: ~57.0 s and ~ 9.12% vs. LL‐BFR‐RT: ~424 s and ~ 81.7% vs. HL‐RT: ~36.0 s and ~ 8.99%, *p* ≤ 0.002). Our results suggest that BFR training performed to failure imposes greater arterial and venous stress in the exercising limb compared to high‐load training without BFR, particularly due to prolonged exposure to elevated pressures. Further research is needed to assess the potential risks of elevated local arterial and venous pressure responses by frequent BFR use, particularly in populations with pre‐existing medical conditions.

## 1 Introduction

Physical training induces a cardiovascular response, typically illustrated by increases in blood pressure (BP) and heart rate (HR). In traditional strength training, the arterial (AP) and venous blood pressure (VP) responses are primarily determined by the number of sets, repetitions, and the mechanical load applied [1]. Depending on the resistance, there is a contraction‐induced compression of intramuscular blood vessels, which leads to an increase in peripheral vascular resistance. In addition, the accumulating metabolites further enhance the cardiovascular response with increasing exercise intensity by activating chemosensitive receptors [2].

In recent years, the Blood Flow Restriction (BFR) training technique has attracted increasing scientific and clinical interest, as significant muscle adaptations can be achieved even with low mechanical loads when venous occlusion is simultaneously induced by externally applied cuffs [3]. This technique introduces a new aspect, a new adjusting variable in training methodology, the externally applied pressure to reduce arterial supply and venous occlusion. Within this regard, BFR training showed significantly higher exercise‐induced rises in AP and VP compared to a control load with the same weight [4, 5]. This demonstrates that the subjective physical exertion, which is significantly higher with BFR training than with comparable interventions, even with light loads, also has a significant effect on the BP response [6]. For this reason, there is already a growing debate about whether this training method can be considered safe [4], especially in clinical patient populations [7].

Comparing strength exercises with different loads, low‐load resistance training (LL‐RT, 30% 1RM) has been shown to be associated with an increase in AP of up to 155/99 mmHg (MAP 113 mmHg) [8], whereas high‐load resistance training (HL‐RT, > 75% 1RM) exercises induce values of up to 480/350 mmHg (MAP 114–212 mmHg) [9], especially during peak loads in exercises such as squat or bench press. The values reported for low‐load BFR resistance training (LL‐BFR‐RT) are between LL‐RT and HL‐RT, with an AP of up to 182/105 mmHg (MAP: 127 mmHg) [8]. Regarding the local VP responses in the exercising limb, it is known that LL‐BFR‐RT leads to a significantly greater exercise‐induced increase in VP compared to a low‐load equivalent control condition [10].

However, these published results should be interpreted with caution, as their conclusions may not fully apply without limitations to the responses of the arterial and venous systems to the BFR method in both healthy and diseased populations. This is basically due to two reasons: (1) the selected measurement method; (2) the applied workload profile (external load). Regarding the first, there are many different ways of measuring BP. A distinction must be made between non‐invasive methods (auscultatory‐, oscillometry‐, photoplethysmogram method) and invasive methods. While non‐invasive methods partially determine the BP indirectly via predefined algorithms (mainly suitable for resting conditions) or rely on measurements on remote extremities, demonstrating the systemic BP responses to exercises [11], these data do not illustrate what happens locally in the exercising, occluded extremity.

Secondly, within most scientific studies on BFR training, a volume‐matched protocol is used to compare exercise approaches. The external load is standardized, usually with the most frequently published exercise protocol, consisting of four sets of 75 repetitions (30‐15‐15‐15), a mechanical load of 30% of the individual one‐repetition maximum (1RM), and, ideally, an individualized, pneumatically regulated BFR pressure application. However, this protocol is unsuitable for drawing valid conclusions about the differences between LL‐RT and LL‐BFR‐RT, as it represents a fundamentally flawed comparison of training intensities. The responses in AP and VP measured under LL‐BFR‐RT mostly correspond to complete physical exhaustion of the subject for the exercise tested, whereas this is not even approximately the case under LL‐RT conditions [12]. For this reason, conclusions regarding BP responses to BFR training can only be drawn if vascular responses to BFR exercise are examined using a methodologically rigorous and validly comparable study design, employing the most appropriate analysis technique, and compared to control conditions.

For this reason, the present study focused on the local physiological reactions of the arterial and venous system to exercise of the elbow flexors using invasive, direct intravascular examinations. Furthermore, in order to reduce internal bias and increase comparability of the intravascular reactions, the external influencing factors of the training were adjusted so that all trials under the conditions LL‐, LL‐BFR‐, and HL‐RT were carried out until voluntary muscle fatigue in a cross over approach.

## 2 Material and Methods

### 2.1 Subjects

Ten healthy male subjects (age: 26.8 ± 4.59 years, height: 171 ± 7.60 cm, body mass: 79.0 ± 8.00 kg) volunteered for this study (Table 1). All subjects were experienced in resistance training (at least twice per week for at least 2 years), had no prior experiences with BFR‐Training, and reported not having performed regular strength training 1 week before the start of the study. Subjects were informed about the experimental procedures and possible risks and signed an informed consent document before the investigation. The study was approved by the local Ethics Committee of the University Hospital Duesseldorf (Trial‐ID: 2015104498) and was performed according to the Declaration of Helsinki.

**TABLE 1:** Classification of diastolic (diasBP), systolic (sysBP), mean arterial (MAP), and peripheral venous (PVP) pressure.

	diasBP	sysBP	MAP	PVP
Zone 1 [mmHg]	< 80	< 120	< 93	< 25
Zone 2 [mmHg]	80–84	120–129	93–99	25–49
Zone 3 [mmHg]	85–89	130–139	100–106	50–74
Zone 4 [mmHg]	90–99	140–159	107–119	75–99
Zone 5 [mmHg]	100–109	160–179	120–132	100–124
Zone 6 [mmHg]	≥ 110	≥ 180	≥ 133	≥ 125

### 2.2 Study Design

To investigate the effects of exercises with different mechanical loadings and the additional application of the BFR technique on intravascular AP and VP, a randomized cross‐over design was applied. The 10 subjects performed an elbow flexor exercise protocol with different mechanical loads and with or without additional BFR at three different time points. In order to reduce the impact of the repeated bout effect [13], the trials were separated by 4 weeks of rest. The participants were randomized via a random‐number table to start with either a low‐load‐(LL‐RT) (*n* = 4), low‐load‐BFR‐ (LL‐BFR‐RT) (*n* = 3) or high‐load resistance exercise session (HL‐RT) (*n* = 3). Therefore, all subjects reported to the laboratory for four testing sessions and follow‐up measurements.

**FIGURE 1:**
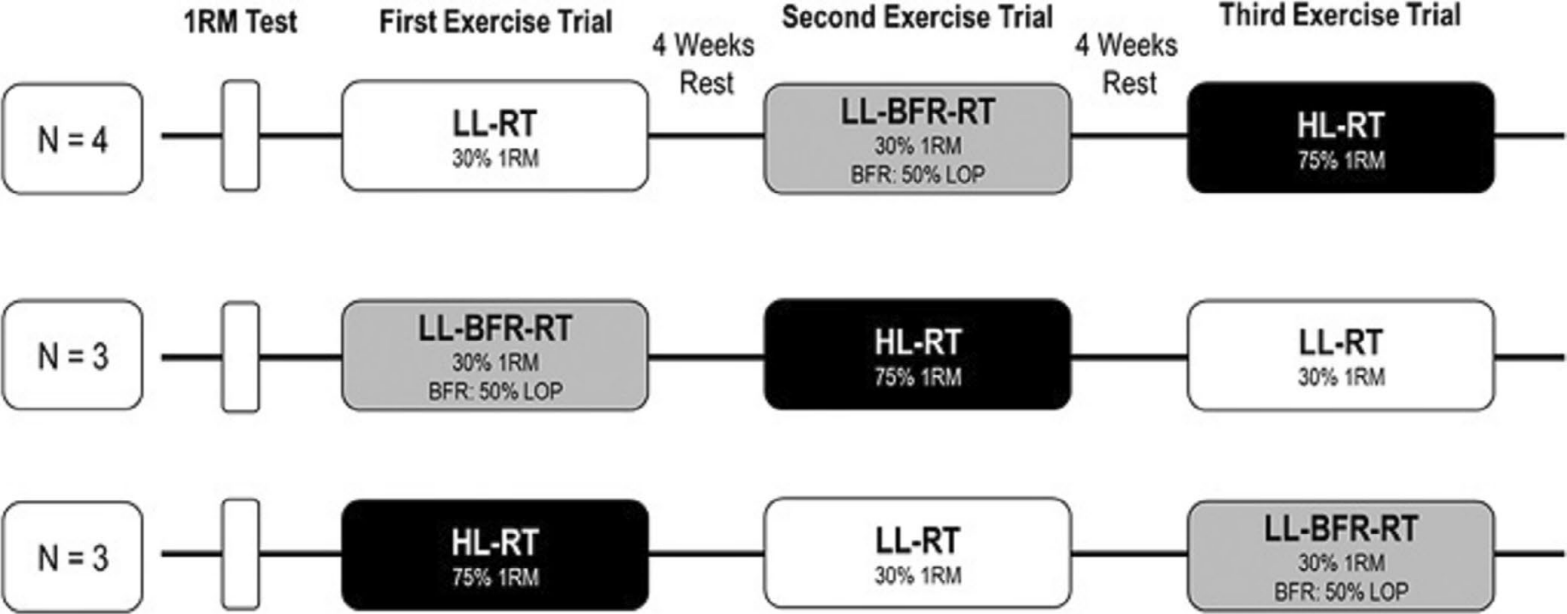
Study design. 1RM = one‐repetition‐maximum, LOP = limb‐occlusion‐pressure, HL‐RT = high‐load resistance exercise session, LL‐BFR‐RT = low‐load‐blood‐flow‐restrcition resistance exercise session, LL‐RT = low‐load resistance exercise session.

During the first visit, subjects' individual concentric 1RM of the elbow flexor of the dominant arm was determined with a dumbbell in accordance to Jessee et al. [14]. Briefly, participants completed 6–8 unilateral elbow flexion (bicep curl) attempts, beginning with an estimated load of 60%–75% of their maximum. During the test, participants were asked to stand against a wall with their elbow fully extended, forearm supinated, and the opposite arm positioned behind the back to minimize extraneous body movement. The load was progressively increased with each attempt until the participant was unable to lift a load greater than their previous successful attempt. A 2‐min recovery period was provided between attempts. The heaviest weight successfully lifted with a full range of motion was defined as the 1RM. After 2 weeks of rest, the second visit was performed by carrying out the experimental loading for the first time. With a 4‐week interval between each following examinations, the other experimental loadings were performed as cross‐over (Figure 1).

### 2.3 Sample Size Calculation

Based on previous studies on the changes in intravascular AP and VP during BFR training [10, 15], we assumed that venous pooling leads to a moderate to high effect size (*f* = 0.35) with respect to our main outcomes. To determine the necessary sample size, we conducted a power analysis using G*Power (version 3.1.9.7). With assumptions of a mean effect size (*f*) of 0.35, an alpha error (ɑ) of 0.05, and statistical power (1 − *β*) of 0.90, the analysis determined a minimum sample size of *N* = 7 for a repeated measures design (within‐between interaction, correlation level among repeated measures: 0.50, non‐sphericity correction: 1.0). Consequently, the attained sample size of *N* = 10 is considered more than adequate to evaluate the study hypothesis.

### 2.4 Interventions

The exercise protocol consisted of unilateral biceps curls with the dominant arm by using a dumbbell (weight scaling: 0.25 kg, ScSports, Emmerich, Germany). All experimental loadings contained a four‐set protocol, where all sets were performed to volitional muscle failure. Following each exercise set, a 60‐s rest period was provided. The key data of the different experimental loading protocols were: LL‐RT: 30% of the 1RM; LL‐BFR‐RT: 30% of the 1RM, 50% of the individual arterial limb‐occlusion‐pressure (LOP); HL‐RT: 75% of the 1RM. Subjects were standing in an upright position, with the back leaning against a wall. Throughout the execution of the exercise, the elbow continuously held contact with the wall. For each repetition, the dumbbell was lifted to a full elbow flexion (~50°). The duration of each repetition was set to 4 s, 2 s for the concentric as well as for the eccentric phase, with no static transition period between contractions. The tempo was controlled by a metronome (60 beats per min). The set was stopped when voluntary muscle failure occurred, which was characterized by the subject being unable to maintain the pace. Based on the performed repetitions in each set, the total workload (repetitions × applied mechanical load [kg]) was determined for comparison between exercise trials. While we did not implement direct physiological monitoring to control for Valsalva maneuvers, no subject exhibited breath‐holding or obvious signs of a Valsalva maneuver during the interventions.

To assess the necessary pressure for the BFR trial, the LOP was determined before the training session with an inflatable tourniquet of 11.5 cm width which was placed proximal at the exercising arm (PBFR, Delfi medical Inc., Vancouver, Canada). After a 10‐min rest period, LOP was determined in a lying position pneumatically by the device and sonographically controlled by displaying the radial artery with an ultrasound device and using a Doppler to assess the blood flow within the vessel. Subsequently, the cuff was inflated until no further blood flow was detectable. This pressure was defined as the individual LOP. The cuff was inflated using a pneumatic cuff inflator (Delfi Medical Inc., Vancouver, Canada) before the start of the LL‐BFR‐RT trial, maintained throughout the exercise period, and deflated immediately after the fourth rest period (i.e., 1 min after completing the exercise protocol).

### 2.5 Local Arterial and Venous Pressure Monitoring

For intravascular pressure monitoring, an arterial and a venous catheter were placed into the dominant arm before exercise. For this purpose, subjects were placed in a lying position on a standard medical examination table. Under local anesthesia with lidocaine hydrochloride, Seldinger's technique was used to puncture the radial artery for arterial access and a dorsal hand vein (Rete venosum dorsale manus) for venous access, respectively. For the determination of intravascular pressures, sensor needles (20 gauge) linked to a line containing a continuous column of saline connected to a transducer unit were used (Infinity Acute Care System, Dräger, Lübeck, Germany). The system was calibrated outside the tissue at the patient's heart level in a standing position. The following parameters were determined every 10 s automatically by using a software analyzing system (e‐data‐grabber, Dräger, Lübeck, Germany): systolic blood pressure (sysBP), diastolic blood pressure (diasBP), mean arterial pressure (MAP) and peripheral venous pressure (PVP).

To assess changes in intravascular pressure during the exercise session, all pressure data were averaged across the following time periods: before exercise (Baseline), each exercise set (Sets 1, 2, 3, and 4), each rest period (Rests 1, 2, 3, and 4), and the 5‐min post‐exercise rest period (Post 5). To further illustrate the pressure response of each experimental protocol, pressure distribution and concentration profiles were constructed. The distribution profile depicts the cumulative time spent above each pressure value, while the concentration profile represents the time spent at each pressure value during the exercise session (see Kosmidis and Passfield [16] for more details on the two analyses). For comparative analysis between experimental protocols, we additionally evaluated time spent within specific pressure zones (Table 1) during the training session, expressed as both absolute duration and relative proportion of the total exercise time. Furthermore, peak values for each intravascular pressure parameter were captured at any time point during the exercise session to reflect the highest cardiovascular load imposed by each experimental protocol.

### 2.6 Statistics

Statistical analysis was performed using R (version 4.2.2). Homoscedasticity and the normal distribution of the data were visually assessed using residual and Q‐Q plots. Linear mixed models (*lme4* package) were used to compare [1] changes in measures over time (fixed effect with 3–8 levels), and [2] differences in time spent (both absolute duration and relative proportion of the total training time) within specific pressure zones (fixed effect with 6 levels) among exercise trials (fixed effect with 3 levels, i.e., LL‐RT vs. LL‐BFR‐RT vs. HL‐RT). To account for inter‐ and intra‐individual variability, each participant's baseline measure was included as a random effect (i.e., random intercept). For total workload and peak intravascular pressure data, significant main effects were explored using a mixed‐effects model with one‐way ANOVA, with participants as a random effect factor and condition as a factor to assess differences between the exercise trials. In case of a significant main and/or interaction effect, multiple pairwise post hoc comparisons with Bonferroni correction were performed (*emmeans* package) to determine which factor levels differ significantly from one another. For all results, an alpha level of 0.05 was interpreted as statistically significant. Data are expressed as the mean ± standard deviation.

## 3 Results

The mean 1RM arm curl and LOP were 16.8 ± 3.90 kg and 165 ± 17.4 mmHg, respectively. The number of repetitions and total workload performed during each exercise trial is summarized in Table 2. Across all exercise sets, participants consistently completed significantly more repetitions in LL‐RT compared to both LL‐BFR‐RT and HL‐RT (*p* < 0.05 and *p* < 0.001, respectively). When comparing LL‐BFR‐RT and HL‐RT, significant differences in the number of repetitions were observed only during Set 1 (*p* < 0.001). Consequently, participants achieved the highest total number of repetitions in LL‐RT compared to both LL‐BFR‐RT and HL‐RT (*p* < 0.001 for both). Furthermore, the number of total repetitions was significantly higher in LL‐BFR‐RT compared to HL‐RT (*p* < 0.001).

**TABLE 2:** Repetitions and total workload during the four sets of low‐load resistance exercise (LL‐RT), LL‐RT with blood flow restriction (LL‐BFR‐RT), and high‐load resistance exercise (HL‐RT).

	LL‐RT	LL‐BFR‐RT	HL‐RT	*p* values from mixed effect model		
				Time	Condition	Time × condition
Repetitions [*n*]						
Set 1	68.8 ± 21.1^a^, ^b^	38.2 ± 6.68^a^	11.5 ± 2.68	< 0.001	< 0.001	< 0.001
Set 2^c^, ^d^	22.1 ± 9.17^a^, ^b^	11.6 ± 3.10	5.20 ± 1.62			
Set 3^c^, ^d^	17.7 ± 6.91^a^, ^b^	9.70 ± 3.02	3.60 ± 1.17			
Set 4^c^, ^d^, ^e^	15.6 ± 6.52^a^, ^b^	9.10 ± 3.57	3.20 ± 1.03			
Total	124 ± 39.8^a^, ^b^	68.6 ± 12.0^a^	23.5 ± 4.70	—	< 0.001	—
Total workload [kg]	692 ± 25.1^a^, ^b^	378 ± 58.7	327 ± 65.1	—	< 0.001	—

*Note:* Data are expressed as the mean ± standard deviation.

^a^ *p* < 0.001, difference to HL‐RT.

^b^ *p* < 0.05, difference to LI‐BFR‐RT.

^c^ *p* < 0.001, difference to Set 1 within LL‐RT.

^d^ *p* < 0.001, difference to Set 1 within LL‐BFR‐RT.

^e^ *p* < 0.05, difference to Set 1 within HL‐RT.

Participants exhibited the highest total workload in the LL‐RT trial compared to both LL‐BFR‐RT and HL‐RT (p < 0.001 for both), with no difference between the LL‐BFR‐RT and HL‐RT trials (*p* = 1.00, Table 2).

**FIGURE 2:**
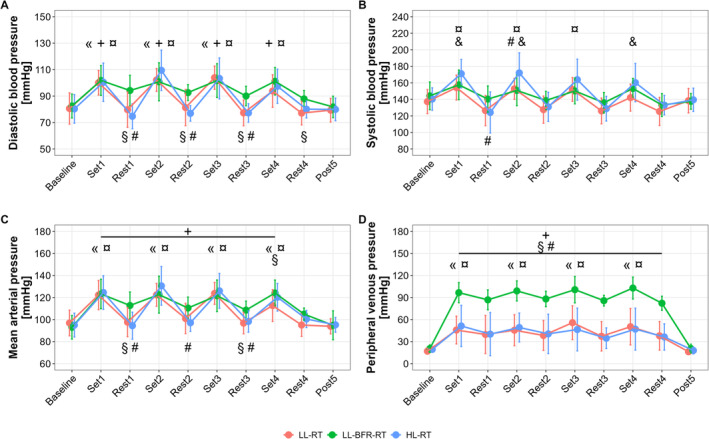
Intravascular pressure during low‐load resistance exercise (LL‐RT), LL‐RT with blood flow restriction (LL‐BFR‐RT), and high‐load resistance exercise (HL‐RT). (A) diastolic blood pressure, (B) systolic blood pressure, (C) mean arterial pressure, (D) peripheral venous pressure. ^«^Significantly different from baseline within LL‐RT (*p* < 0.05), ^+^Significantly different from baseline within LL‐BFR‐RT (*p* < 0.05), ^¤^Significantly different from baseline within HL‐RT (*p* < 0.05), ^§^significant difference between LL‐RT and LL‐BFR‐RT within the respective time point (*p* < 0.05), ^#^significant difference between HL‐RT and LL‐BFR‐RT within the respective time point (*p* < 0.05), ^&^significant difference between LL‐RT and HL‐RT within the respective time point (*p* < 0.05).

Significant main effects for time (*p* < 0.001), condition (*p* < 0.001), and their interactions (*p* ≤ 0.006) were found for all intravascular pressure parameters on average (Figure 2). Across all trials, diasBP (Figure 2A) was significantly elevated during each exercise set compared to baseline levels (*p* < 0.001, except for LL‐RT at Set 4, *p* = 0.073), with no significant differences observed among conditions. During rest periods, the diasBP levels in LL‐BFR‐RT were significantly higher than in other exercise conditions (*p* ≤ 0.02, except for HL‐RT at Rest 4, *p* = 0.184). However, these diasBP levels in LL‐BFR‐RT did not differ significantly from the baseline level (*p* ≥ 0.232). For sysBP (Figure 2B), HL‐RT showed a more pronounced increase during each exercise set (compared to baseline, *p* ≤ 0.01, except in Set 4, *p* = 0.084), with significantly higher values compared to LL‐RT during Sets 1, 2, and 4 (*p* ≤ 0.03) and compared to LL‐BFR‐RT during Set 2 (*p* = 0.003). Whereas LL‐BFR‐RT showed significantly higher MAP and PVP values (Figure 2C,D) during both exercise and rest periods compared to the baseline level (*p* ≤ 0.02, except for MAP in Rest 4, *p* = 0.298), the LL‐RT and HL‐RT protocols resulted in a significant increase in MAP and PVP only during exercise sets (*p* ≤ 0.02). Consequently, significant differences in MAP were observed between LL‐BFR‐RT and other exercise conditions during rest periods (*p* ≤ 0.05, except in Rest 4, *p* ≥ 0.073), but not during exercise periods (*p* ≥ 0.206, except for LL‐RT in Set 4, *p* = 0.022). Regarding PVP, LL‐BFR‐RT demonstrated significantly higher values compared to other exercise conditions during both exercise and rest periods (*p* < 0.001). Following 5 min of recovery after the exercise session, all intravascular pressure parameters returned to baseline levels (*p* = 1.00) regardless of the exercise protocol performed.

**FIGURE 3:**
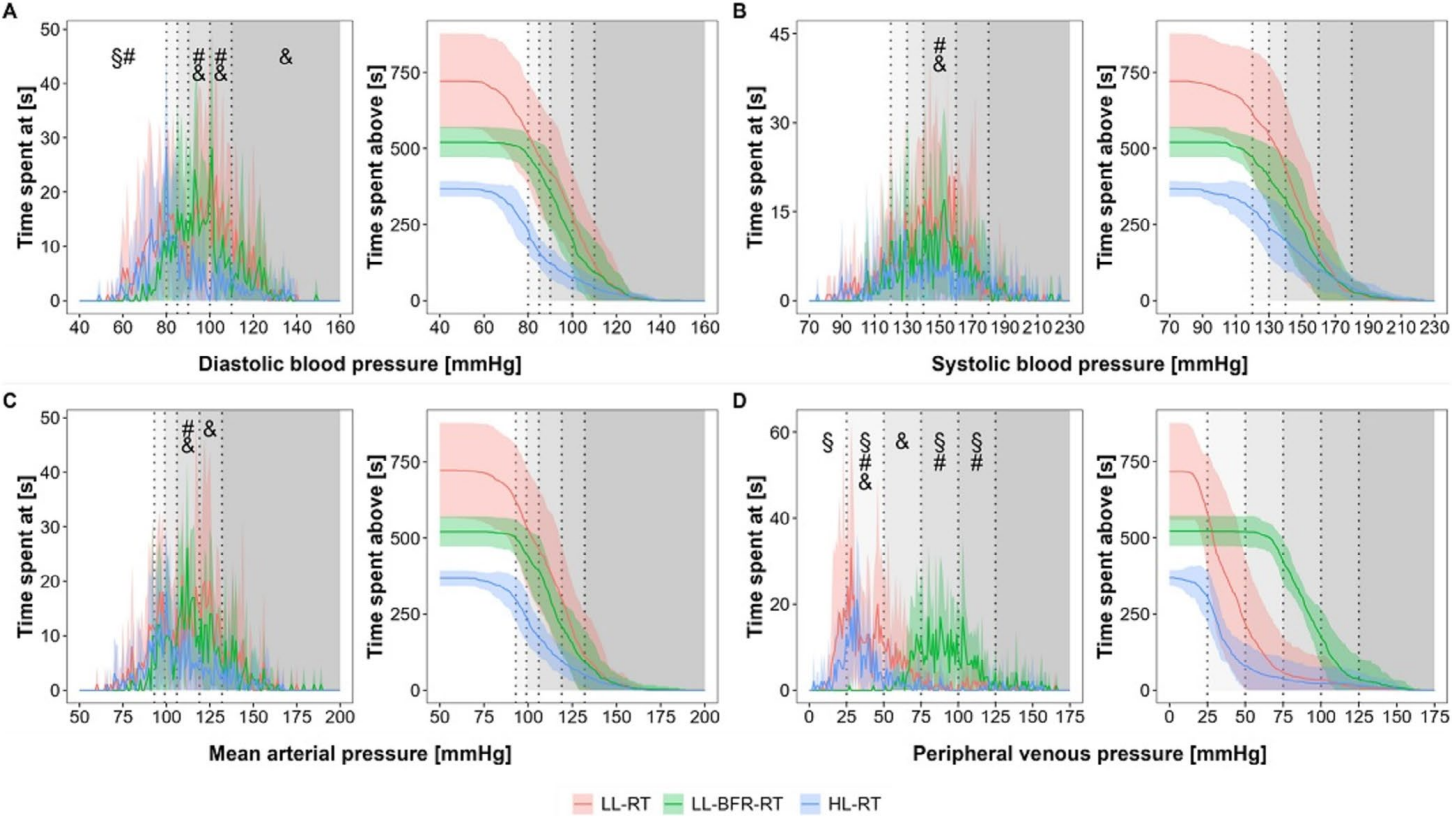
Intravascular pressure concentration (left) and distribution (right) profiles expressed as absolute duration [s] during low‐load resistance exercise (LL‐RT), LL‐RT with blood flow restriction (LL‐BFR‐RT), and high‐load resistance exercise (HL‐RT). (A) diastolic blood pressure, (B) systolic blood pressure, (C) mean arterial pressure, (D) peripheral venous pressure. Data are presented as mean values (line plots) with standard deviations (shaded areas). The gray‐shaded areas in the background indicate the specific zones for each intravascular pressure parameter (see Table 1). ^§^significant difference between LL‐RT and LL‐BFR‐RT within the respective pressure zone (*p* < 0.05), ^#^significant difference between HL‐RT and LL‐BFR‐RT within the respective pressure zone (*p* < 0.05), ^&^significant difference between LL‐RT and HL‐RT within the respective pressure zone (*p* < 0.05).

**FIGURE 4:**
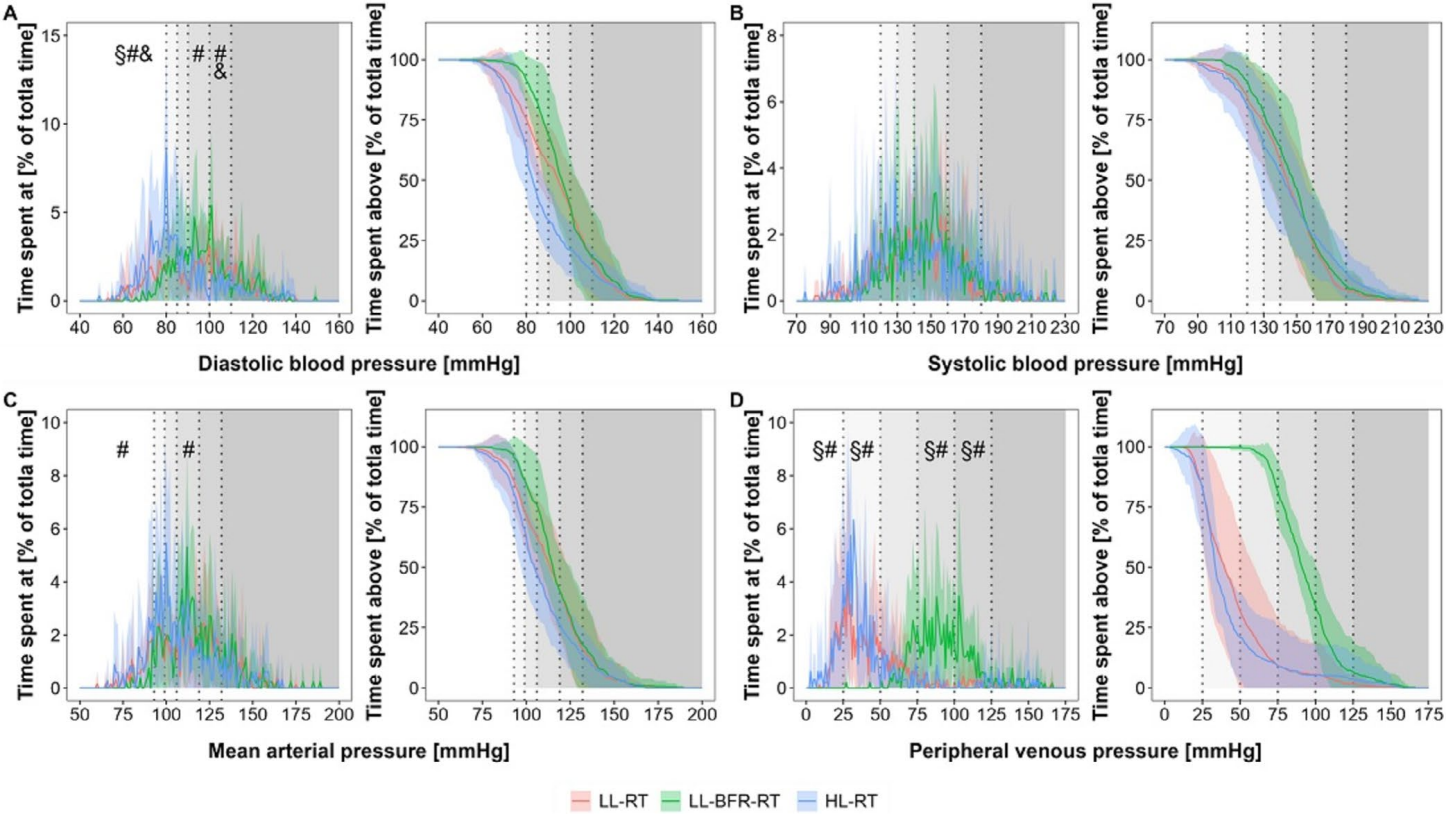
Intravascular pressure concentration (left) and distribution (right) profiles expressed as relative proportion of the total training time during low‐load resistance exercise (LL‐RT), LL‐RT with blood flow restriction (LL‐BFR‐RT), and high‐load resistance exercise (HL‐RT). (A) diastolic blood pressure, (B) systolic blood pressure, (C) mean arterial pressure, (D) peripheral venous pressure. Data are presented as mean values (line plots) with standard deviations (shaded areas). The gray‐shaded areas in the background indicate the specific zones for each intravascular pressure parameter (see Table 1). ^§^significant difference between LL‐RT and LL‐BFR‐RT within the respective pressure zone (*p* < 0.05), ^#^significant difference between HL‐RT and LL‐BFR‐RT within the respective pressure zone (*p* < 0.05), ^&^significant difference between LL‐RT and HL‐RT within the respective pressure zone (*p* < 0.05).

Figures 3 and 4 depict the intravascular pressure concentration and distribution profiles expressed as an absolute duration and relative proportion of total training time, respectively.

For the absolute time spent within specific pressure zones (concentration profiles in Figure 3), significant main effects were observed for condition (*p* ≤ 0.001), pressure zone (*p* < 0.001), and their interaction (*p* ≤ 0.022) across all intravascular pressure parameters. Further analysis indicated that both LL‐RT and LL‐BFR‐RT resulted in significantly more time spent at higher diasBP (90–109 mmHg; LL‐RT: 298 ± 188 s, LL‐BFR‐RT: 265 ± 76.3 s), sysBP (140–159 mmHg; LL‐RT: 272 ± 186 s, LL‐BFR‐RT: 206 ± 79.5 s), and MAP levels (107–132 mmHg; LL‐RT: 346 ± 207 s, LL‐BFR‐RT: 296 ± 81.9 s) compared to HL‐RT (diasBP: 76.0 ± 28.4 s, sysBP: 96.0 ± 57.0 s, MAP: 122 ± 43.9 s; *p* ≤ 0.028 for all). For PVP, LL‐BFR‐RT showed a significantly greater absolute time at higher pressure levels (75–125 mmHg; 387 ± 117 s) compared to the other exercise conditions (LL‐RT: 47.0 ± 90.6 s, HL‐RT: 21.0 ± 43.3 s; p ≤ 0.001).

Regarding the relative proportion of total training time spent within specific pressure zones (concentration profiles in Figure 4), no condition effects (*p* ≥ 0.998), but significant main effects for pressure zone (*p* < 0.001) and interaction effects (*p* ≤ 0.004, except for sysBP, *p* = 0.195) were found for all intravascular pressure parameters. Further analysis revealed that participants spent a significantly greater proportion of total training time at higher diasBP (90–109 mmHg) and MAP levels (140–159 mmHg) during LL‐BFR‐RT (50.9% ± 14.0% and 35.1% ± 11.3%, respectively) compared to HL‐RT (diasBP: 20.9% ± 8.03%, MAP: 20.8% ± 13.1%; *p* ≤ 0.034), but not compared to LL‐RT (diasBP: 39.1% ± 16.8%, MAP: 22.5% ± 10.6%; *p* ≥ 0.069). Additionally, LL‐BFR‐RT led to a significantly higher proportion of time spent at elevated PVP (75–125 mmHg; 74.8% ± 22.4%) compared to both LL‐RT and HL‐RT (7.60% ± 15.3% and 5.42 ± 11.1, respectively; *p* ≤ 0.002).

The absolute duration and relative proportion of total training time spent within specific pressure zones for each exercise condition are provided in the supplemental material.

No significant differences were observed in peak values of diasBP, sysBP, and MAP between exercise conditions (Table 3). However, peak PVP was significantly higher during LL‐BFR‐RT compared to both LL‐RT and HL‐RT (*p* < 0.05).

**TABLE 3:** Peak values of intravascular pressure measured at any time points during low‐load resistance exercise (LL‐RT), LL‐RT with blood flow restriction (LL‐BFR‐RT), and high‐load resistance exercise (HL‐RT).

	LL‐RT	LL‐BFR‐RT	HL‐RT	*p* values from mixed effect model
diasBP [mmHg]	121 ± 10.4 (105–140)	122 ± 14.3 (105–149)	123 ± 13.3 (104–139)	0.904
sysBP [mmHg]	181 ± 18.9 (159–214)	186 ± 24.0 (158–224)	148 ± 13.1 (123–167)	0.297
MAP [mmHg]	148 ± 17.1 (125–175)	146 ± 19.0 (127–189)	148 ± 13.1 (123–167)	0.931
PVP [mmHg]	87.8 ± 48.3 (36.0–165)	125 ± 20.0^a^, ^b^ (100–166)	80.0 ± 34.9 (48–157)	0.006

*Note:* Data are expressed as the mean ± standard deviation (min—max).

^a^ *p* < 0.05, difference to LI‐RT.

^b^ *p* < 0.001, difference to HL‐RT.

## 4 Discussion

The present study is the first to describe the acute AP and VP responses to LL–, LL–BFR–, and HL–RT of the elbow flexors, continuously monitored during exercise sets and in–between rest periods using invasive catheter measurements locally in the exercising arm. Within the comparison of the investigated conditions, LL–BFR–RT shows significantly higher diasBP and MAP values during the rest periods. The corresponding concentration profile illustrates that with using the BFR technique, the diasBP is significantly longer (in total and relative time) in pressure zones above 80 mmHg compared to LL– or HL–RT. Interestingly, whereas the peak values of sysBP, diasBP, and MAP did not differ between the trials, both LL conditions show significantly longer periods in higher pressure zones than under HL–condition. Furthermore, the PVP induced by BFR is consistently (across all sets and rests) significantly higher than the comparative trials, with longer time in pressure zones beyond 75 mmHg. The present data show for the first time that when the muscles of the elbow flexors are exercised to fatigue, local arterial and venous stress during LL– and LL–BFR–RT is higher than during HL–RT.

### 4.1 Arterial Responses to Blood–Flow–Restriction

For the first time, this project was able to provide a continuous measurement of arterial and venous responses within the exercising limb, highlighting the differences between the three different modes of exercise. While it was previously assumed that the AP responses during BFR exercise fell between those observed during HL–RT and LL–RT, the present data suggest a different conclusion within the framework of a unilateral elbow flexor protocol. While HL–RT showed on average higher local sysBP values during exercise only in comparison to LL–RT, the LL–BFR–RT trial shows significantly higher diasBP and MAP values within all rest periods (Figure 2). These results are surprising as previously published studies about systemic BP responses have shown higher BP values during exercises under HL conditions [17] or during BFR exercise within pre to post set comparisons [10]. Therefore, the present local results challenge the conceived paradigm of cardiovascular reactions HL>LL–BFR>LL–RT [18]. However, potential explanations for these results may be found in the examination method itself, the measurement location, and the applied protocol. Typically, non–invasive techniques are used to analyze cardiovascular responses to exercise interventions. Within this regard, the often applied auscultatory method for measuring BP is criticized for its invalidity due to factors like inaccurate equipment, inadequate training, evaluator inexperience, and for not being able to measure systolic and diastolic pressure simultaneously [19]. The oscillometry method on the other hand, used in most automatic devices, estimates BP accurately at rest but fails during exercise due to changes in systolic and diastolic time intervals [20]. In addition, both techniques do not allow the analysis of pressure responses at the location of the exercise, only remotely from the exercising limb or in–between sets, therefore representing more systemic adaptations than local.

To the best of the authors' knowledge, only one study of our research group used the same invasive technique to obtain local AP responses to exercise before [10]. Within this study, we found that by using a volume–matched exercise protocol of the elbow flexors, the sysBP was significantly higher at the end of each exercising set and the diasBP was continuously increased during the rest periods by using LL–BFR–RT in comparison to LL–RT alone. These findings are consistant with the literature, illustrating that volume–matched protocols regularly show higher rises in sysBP and diasBP after exercise sets with LL–BFR– compared to LL–RT [18]. However, this comparison is not valid. Although the external load characteristics (repetitions, mechanical load, rest time) are usually standardized in volume–matched protocols, the applied BFR pressure and its impact on subjective exercise intensity is rarely taken into account. For this reason, subjects under BFR conditions are exposed to a greater stress within this comparison, which consequently results in a higher BP response. This conclusion is confirmed by the present results, which show that if all exercise loads are carried out until voluntary muscle fatigue, there are no differences in the pre– and post–exercise data (Figure 2) or even in the peak pressure data (Table 3).

### 4.2 Continuous Monitoring and Concentration Profiles

However, comparisons of pressure responses between sets do not reflect the actual physiological reaction of the body during exercise, rather they only represent single time points. Within this regard, Brandner et al. [17] were able to show in unilateral biceps curl exercise, that in comparison between HL–RT (80% 1RM, 4× 6–8 reps) and LL–BFR–RT (20% 1RM, LOP: 80% of the sysBP, non–regulated device, 30–15–15–15 reps) significantly higher systemic values for sysBP, diasBP, and MAP were obtained on the non–exercised arm via manual auscultation during the sets. Direct, intravascular measurements in the present fatiguing protocol showed a similar result only during set two in comparison to both LL–conditions. While the continuous measurement of AP during the sets and rests revealed no further differences in terms of a higher response due to HL–RT, the rest periods in particular showed significantly increased diasBP and MAP values in the BFR–trial.

Higher resting diasBP in systemic measurements with LL–BFR–RT were previously described in comparison to HL [18] and LL control conditions [10]. These could mainly be attributed to rising pressure in the arterial vessels if the cardiac output is pushed back against the stasis (i.e., arterial pooling), or by longer exercise times, respectively longer times under an external stressor (if BFR is continuously applied during rest period as well) and the associated longer activation of the sympathetic nervous system [21]. The latter statement is initially supported by the calculated workload (Table 2) and corresponding durations of the performed sets (Supporting Information, Tables 1 and 2) and is illustrated in the concentration profiles of the absolute training times of the investigated conditions as well (Figure 3).

The concentration profiles created show how much absolute time of the training was spent in a defined local pressure zone (using the classification of [22]). A visualization and statistical analysis of the pressure values in this way shows that the two load patterns LL–RT and LL–BFR–RT are significantly longer in sysBP zones between 140 and 160 mmHg. A comparison of the diasBP revealed a similar outcome, illustrating that the low‐load exercises spend significantly more time in higher pressure zones (90–99 and 100–109 mmHg), and that the two forms of exercise without BFR spend significantly more time in diasBP values below 80 mmHg, especially during rest periods. A similar pattern is also reflected by the MAP, which shows that LL–RT and LL–BFR–RT spend significantly longer in the 107–119 mmHg pressure zone than the HL control conditions. In summary, the concentration profile of the absolute times of the three forms of exercise shows that the significantly longer training times of the low–load conditions also result in considerably higher exercise durations in significantly higher sysBP, diasBP, and MAP zones.

However, to put the training times in different pressure zones into perspective, Figure 4 shows the concentration profiles of the three types of training with relative time. Here, a similar pattern was observed with a significantly higher percentage of training time in the LL–RT and LL–BFR–RT conditions in higher pressure zones of diasBP and MAP. Therefore, it can be concluded that if an exercise session is carried out with low mechanical loads up to volitional muscle fatigue, with and without the additional BFR application, significantly longer times (absolute and relative) are spent in higher diasBP and MAP zones than in training with high mechanical loads.

### 4.3 Venous Response to Blood–Flow–Restriction Training

The venous system represents a fairly under–researched area in the field of exercise sciences. In a volume–matched protocol of the elbow flexors, LL–BFR–RT shows significantly higher local VP values at the wrist than LL–RT up to around 65 mmHg [10]. In the present study, the use of external occlusion within a fatiguing exercise protocol almost doubled VP to 125 ± 20.0 mmHg (*p* < 0.05). Even though the control groups without external occlusion show a significant increase compared to the resting value, the values within the BFR trials are considerably higher and are statistically longer in the high‐pressure zones 75–125 mmHg (Figures 3 and 4).

### 4.4 Limitations

The present work and the applied protocol also show limitations that influence the results. First, the study population consisted exclusively of healthy young men with experience in resistance training. While this ensured comparability with our previous research [10, 15] and allowed for a more consistent interpretation of results across studies, it limited the generalizability of the findings to women and clinical patient populations. Therefore, future studies including female participants and clinical populations are warranted to evaluate potential sex‐specific differences and enhance the applicability of the findings to a broader and more diverse population. Secondly, the applied measurement technique was limited to a data sampling of 0.1 Hz and represents an average of a 2 s time period. This time delay in recording caused more measurement points to be available for data collection during longer exercises (LL and LL–BFR–RT) than during shorter ones (HL–RT). Thirdly, our findings are exclusively applicable to unilateral upper limb training loads and represent the local, not systemic BP responses to the exercises. However, the present findings can provide an estimate of changes and responses in AP and VP even during multi‐limb or more complex exercises. Furthermore, it was not possible to differentiate between concentric and eccentric exercise phases with this recording frequency. Thus, the pressure measurements were not standardized in a contraction phase. However, as this was the same under all conditions, this disadvantage is the same under all conditions.

In conclusion, the present study demonstrates for the first time that the proposed paradigm for systemic AP responses to exercises (HL>LL–BFR>LL–RT) is probably only applicable in volume–matched exercise comparisons and does not represent BP responses locally in the exercising arm. As the external BFR pressure and its effect on cardiovascular physiology and subjective exercise effort is not taken into account in this comparison, the exercises in the present project were performed until voluntary muscle failure under all conditions. Hereby, we were able to show that the low–load exercise conditions spend significantly longer times (absolute and relative) in higher diasBP and MAP zones than HL exercise, measured in the exercising limb. This result has a far–reaching impact, particularly for the practical application of BFR training.

## 5 Perspective

Our findings challenge the conventional belief that high‐load training imposes the highest intravascular stress. Instead, BFR training performed to failure led to prolonged exposure to elevated pressures compared to high‐load training without BFR in the exercising extremity, mostly due to longer exercise times. These insights have important implications for assessing the intravascular pressure responses and thereby applicability of BFR, particularly in clinical populations. Since the BFR method is able to train also physically impaired patients rapidly and effectively to a state of physical exhaustion, it should be considered that although volume‐matched protocols with low mechanical loads are applied, the cardiovascular response could be similar or even higher than in high–load exercises. Furthermore, the present study was able to show the huge impact of the BFR method on VP responses, leading to significant local venous hypertension. In a recent study on the lower extremity, we could not identify any negative influence of a single BFR application with associated venous hypertension on post–functional venous compliance [15]. However, since this study was an acute study performed in young and healthy men, it remains questionable whether there may be negative effects on venous function if the vessels in the exercising limb are already pre–affected (e.g., venous insufficiency) or if the BFR training technique is used more frequently. Future studies should investigate the influence of BFR training in specific patient cohorts or in regular users phlebodynamometrically in order to uncover a possible risk of the onset or progress of venous pathologies (e.g., venous insufficiency) for use.

## Conflicts of Interest

The authors declare no conflicts of interest.

## Transparency Declaration

The authors affirm that this manuscript is an honest, accurate, and transparent account of the study being reported; that no important aspects of the study have been omitted; and that any discrepancies from the study as planned have been explained.

## Supporting information


Data S1.

## Data Availability

The data that support the findings of this study are available from the corresponding author upon reasonable request. Source data underlying all Figures and Tables are provided as a Source.
