# Supplementary material for: Arterial and Venous Pressure Dynamics in Blood Flow Restriction Versus Traditional Strength Training
Source: Scand J Med Sci Sports. 2025 Feb 17;35(2):e70029. doi: 10.1111/sms.70029 (PMC11832360; doi:10.1111/sms.70029)
Supplement: Supplementary file 1 — Data S1. [file SMS-35-e70029-s001.docx]

| **Variable** | **Condition** | **Time in zone [s]** | | | | | |
| --- | --- | --- | --- | --- | --- | --- | --- |
|  |  | **Zone 1** | **Zone 2** | **Zone 3** | **Zone 4** | **Zone 5** | **Zone 6** |
| diaBP | LL-RT | 174 ± 87.3 | 73.0 ± 56.4 | 56.0 ± 32.7 | 139 ± 95.5^#^ | 159 ± 115^#^ | 121 ± 137^#^ |
|  | LL-BFR-RT | 45.0 ± 51.9^*#^ | 48.0 ± 52.2 | 72.0 ± 54.3 | 153 ± 51.7^#^ | 112 ± 33.9^#^ | 91.0 ± 101 |
|  | HL-RT | 137 ± 70.7 | 72.0 ± 47.8 | 37.0 ± 31.3 | 45.0 ± 25.9 | 31.0 ± 16.6 | 46.0 ± 33.1 |
| sysBP | LL-RT | 103 ± 105 | 76.0 ± 49.0 | 106 ± 59.1 | 272 ± 186^#^ | 137 ± 131 | 28.0 ± 45.9 |
|  | LL-BFR-RT | 47.0 ± 62.7 | 66.0 ± 64.5 | 72.0 ± 60.1 | 206 ± 79.5^#^ | 98.0 ± 84.3 | 32.0 ± 45.7 |
|  | HL-RT | 68.0 ± 58.5 | 63.0 ± 44.7 | 38.0 ± 17.5 | 96.0 ± 57.0 | 61.0 ± 55.9 | 42.0 ± 39.1 |
| MAP | LL-RT | 101 ± 92.8 | 92.0 ± 51.2 | 78.0 ± 48.9 | 167 ± 96.6^#^ | 179 ± 139^#^ | 105 ± 143 |
|  | LL-BFR-RT | 24.0 ± 46.0 | 58.0 ± 57.5 | 55.0 ± 34.1 | 182 ± 59.0^#^ | 114 ± 54.8 | 88.0 ± 102 |
|  | HL-RT | 72.0 ± 64.4 | 65.0 ± 44.8 | 63.0 ± 43.0 | 77.0 ± 49.5 | 45.0 ± 32.4 | 46.0 ± 38.6 |
| PVP | LL-RT | 137 ± 193 | 362 ± 167^#^ | 161 ± 175 | 23.0 ± 38.0 | 24.0 ± 53.8 | 10.0 ± 24.9 |
|  | LL-BFR-RT | 0.00 ± 0.00^*^ | 2.00 ± 6.30^*#^ | 96.0 ± 68.2^*^ | 251 ± 141^*#^ | 136 ± 70.1^*#^ | 37.0 ± 74.5 |
|  | HL-RT | 62.0 ± 79.4 | 227 ± 59.3 | 43.0 ± 34.7^*^ | 14.0 ± 28.0 | 7.00 ± 16.4 | 15.0 ± 47.4 |

**Supplemental Table S1.** Time spent within specific pressure zones during the training session during low-load resistance exercise (LL-RT), LL-RT with blood flow restriction (LL-BFR-RT), and high-load resistance exercise (HL-RT).

Corresponding zones for each intravascular pressure parameter were defined as followed: diastolic blood pressure (diaBP): < 80 mmHg, 80 – 84 mmHg, 85 – 89 mmHg, 90 – 99 mmHg, 100 – 109 mmHg, ≥ 110 mmHg; systolic blood pressure (sysBP): < 120 mmHg, 120 – 129 mmHg, 130 – 139 mmHg, 140 – 159 mmHg, 160 – 179 mmHg, ≥ 180 mmHg; mean arterial pressure (MAP): < 93 mmHg, 93 – 99 mmHg, 100 – 106 mmHg, 107 – 119 mmHg, 120 – 132 mmHg, ≥ 133 mmHg; peripheral venous pressure (PVP): < 25 mmHg, 25 – 49 mmHg, 50 – 74 mmHg, 75 – 99 mmHg, 100 – 124 mmHg, ≥ 125 mmHg); ^*^*p* < 0.01, difference to LL-RT, ^#^*p* < 0.01, difference to HL-RT.

**Supplemental Table S2.** Relative proportion of the total training time within specific pressure zones during the training session during low-load resistance exercise (LL-RT), LL-RT with blood flow restriction (LL-BFR-RT), and high-load resistance exercise (HL-RT).

| **Variable** | **Condition** | **Relative time in zone [%]** | | | | | |
| --- | --- | --- | --- | --- | --- | --- | --- |
|  |  | **Zone 1** | **Zone 2** | **Zone 3** | **Zone 4** | **Zone 5** | **Zone 6** |
| diaBP | LL-RT | 24.6 ± 12.9 | 10.8 ± 8.78 | 7.98 ± 5.38 | 18.5 ± 10.1 | 20.6 ± 10.3^#^ | 17.4 ± 18.8 |
|  | LL-BFR-RT | 8.46 ± 9.80^*#^ | 9.12 ± 9.86 | 13.7 ± 10.3 | 29.4 ± 9.34^#^ | 21.6 ± 6.73^#^ | 17.7 ± 19.4 |
|  | HL-RT | 36.9 ± 18.7^*^ | 19.5 ± 12.6 | 10.1 ± 8.29 | 12.4 ± 7.28 | 8.47 ± 4.52 | 12.6 ± 9.09 |
| sysBP | LL-RT | 15.4 ± 15.6 | 11.5 ± 8.07 | 14.6 ± 6.97 | 35.4 ± 17.6 | 18.7 ± 16.4 | 4.46 ± 7.60 |
|  | LL-BFR-RT | 8.77 ± 11.3 | 13.1 ± 13.0 | 14.0 ± 11.6 | 39.3 ± 14.7 | 18.7 ± 15.3 | 6.29 ± 8.82 |
|  | HL-RT | 18.8 ± 16.2 | 17.4 ± 12.7 | 10.5 ± 5.05 | 26.1 ± 15.6 | 15.9 ± 13.3 | 11.4 ± 10.7 |
| MAP | LL-RT | 14.6 ± 12.9 | 13.5 ± 8.19 | 11.1 ± 6.86 | 22.5 ± 10.6 | 23.1 ± 13.0 | 15.2 ± 19.4 |
|  | LL-BFR-RT | 4.45 ± 8.57^#^ | 11.0 ± 10.9 | 10.6 ± 6.55 | 35.1 ± 11.3^#^ | 21.7 ± 10.1 | 17.2 ± 19.9 |
|  | HL-RT | 19.7 ± 17.3 | 17.7 ± 12.4 | 16.9 ± 10.7 | 20.8 ± 13.1 | 12.4 ± 8.92 | 12.5 ± 10.6 |
| PVP | LL-RT | 17.5 ± 21.8 | 50.0 ± 22.8 | 22.7 ± 22.9 | 3.60 ± 6.28 | 3.89 ± 9.09 | 1.63 ± 4.23 |
|  | LL-BFR-RT | 0.00 ± 0.00^*#^ | 0.38 ± 1.22^*#^ | 18.2 ± 12.6 | 48.0 ± 24.1^*#^ | 27.0 ± 14.8^*#^ | 6.71 ± 13.2 |
|  | HL-RT | 17.3 ± 22.6 | 62.1 ± 17.5 | 11.8 ± 9.75 | 3.66 ± 7.20 | 1.76 ± 4.17 | 3.57 ± 11.3 |

Corresponding zones for each intravascular pressure parameter were defined as followed: diastolic blood pressure (diaBP): < 80 mmHg, 80 – 84 mmHg, 85 – 89 mmHg, 90 – 99 mmHg, 100 – 109 mmHg, ≥ 110 mmHg; systolic blood pressure (sysBP): < 120 mmHg, 120 – 129 mmHg, 130 – 139 mmHg, 140 – 159 mmHg, 160 – 179 mmHg, ≥ 180 mmHg; mean arterial pressure (MAP): < 93 mmHg, 93 – 99 mmHg, 100 – 106 mmHg, 107 – 119 mmHg, 120 – 132 mmHg, ≥ 133 mmHg; peripheral venous pressure (PVP): < 25 mmHg, 25 – 49 mmHg, 50 – 74 mmHg, 75 – 99 mmHg, 100 – 124 mmHg, ≥ 125 mmHg); ^*^*p* < 0.01, difference to LL-RT, ^#^*p* < 0.01, difference to HL-RT.
